# The therapeutic effect of larval saliva and hemolymph of *Lucilia sericata* on the treatment of *Leishmania major* lesion in BALB/c mice946

**DOI:** 10.1186/s13071-023-05660-0

**Published:** 2023-02-16

**Authors:** Sara Rahimi, Javad Rafinejad, Amir Ahmad Akhavan, Reza Ahmadkhaniha, Mahmood Bakhtiyari, Ali Khamesipour, Kamran Akbarzadeh

**Affiliations:** 1 Medicinal Plants Research Center, Maragheh University of Medical Sciences, Maragheh, Iran; 2 Department of Medical Entomology and Vector Control, School of Public Health, Tehran University of Medical Sciences, Tehran, Iran; 3 Pharmaceutical Chemistry, Department of Human Ecology, School of Public Health, Tehran University of Medical Sciences, Tehran, Iran; 4 Department of Community Medicine and Epidemiology, School of Medicine Non-Communicable Diseases Research Center Alborz University of Medical Sciences, Karaj, Iran; 5 Center for Research and Training in Skin Diseases and Leprosy, Tehran University of Medical Sciences, Tehran, Iran

**Keywords:** Leishmanial activity, *Lucilia sericata*, *Leishmania major*, Cutaneous leishmaniasis, Glucantime, Natural compound

## Abstract

**Background:**

Treatment of cutaneous leishmaniasis (CL) remains a major challenge for the public health and medical community. It has been claimed that natural compounds derived from fly larvae have anti-leishmania properties against some species of *Leishmania*. The present study aimed at assessing the in vitro effects of larval products of *Lucilia sericata* against the promastigote and intracellular amastigote forms of *Leishmania major*. Also, the therapeutic effect of larval products on lesions induced by *L. major* infection was evaluated in BALB/c mice models.

**Methods:**

Parasite specimens and macrophage cells were exposed to varying concentrations of larval products for 24–120 h. Lesion progression and parasite load were investigated in the models to assess the therapeutic effects of the products.

**Results:**

The larval products displayed more potent cytotoxicity against *L. major* promastigotes. The IC_50_ values for larval saliva and hemolymph were 100.6 and 37.96 ug/ml, respectively. The IC_50_ of glucantime was 9.480 ug/ml. Also, the saliva and hemolymph of *L. sericata* exhibited higher cytotoxicity against the promastigotes of *L. major* but were less toxic to the macrophage cells. Treatment with leishmanicidal agents derived from larvae of *L. sericata* decreased the infection rate and the number of amastigotes per infected host cell at all concentrations. Lesion size was significantly (*F*_(7, 38)_ = 8.54, *P* < 0.0001) smaller in the treated mice compared with the untreated control group. The average parasite burden in the treated mice groups (1.81 ± 0.74, 1.03 ± 0.45 and 3.37 ± 0.41) was similar to the group treated with a daily injection of glucantime (1.77 ± 0.99) and significantly lower (*F*_(7, 16)_ = 66.39, *P* < 0.0001) than in the untreated control group (6.72 ± 2.37).

**Conclusions:**

The results suggest that the larval products of *L. sericata* were effective against *L. major* parasites both in vivo and in vitro. However, more clinical trial studies are recommended to evaluate the effects of these larval products on human subjects.

**Graphical Abstract:**

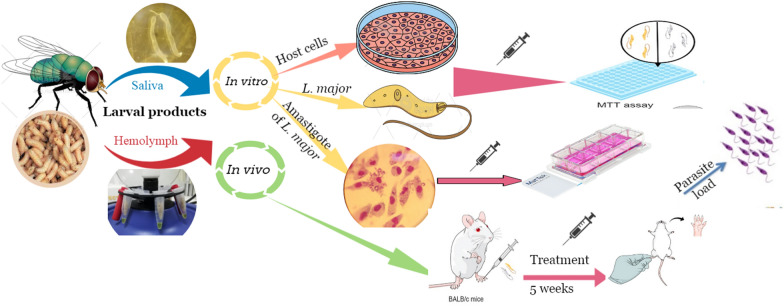

## Background

Leishmaniasis is a group of infectious diseases caused by protozoan parasites of the genus *Leishmania* [1]. Human transmission occurs by the bite of infected female sandflies. The disease exhibits a spectrum of clinical manifestations, ranging from mild asymptomatic forms to pleiomorphic cutaneous and mucocutaneous forms or fatal visceral forms [2, 3].

Leishmaniasis is a major neglected tropical disease (NTD) [4]. The disease primarily affects individuals from underdeveloped and developing countries in Africa, Asia and Latin America. The major risk factors associated with the disease among individuals from these regions include immunodeficiencies, poor nutrition, increased urbanization, poor living conditions and lack of resources [5, 6].

Leishmaniasis endemicity was reported in about 98 countries in 2020, according to the World Health Organization (WHO). Endemic cases of both VL and CL were together reported in more than two-thirds of these countries (71 countries); however, the presence of only VL and only CL was reported in 8 countries and 19 countries, respectively [6]. Annually, about 50,000 to 90,000 new cases of VL and about 600,000 to 1 million new cases of CL are reported worldwide [2].

The study of leishmaniasis in Iran has an age-long history. According to the Islamic Republic of Iran’s Ministry of Health and Medical Education, the annual incidence of CL in Iran is approximately 20,000 cases. Zoonotic cutaneous leishmaniasis (ZCL) lesions induced by *Leishmania major* and anthroponotic cutaneous leishmaniasis (ACL) lesions induced by *Leishmania tropica* are the dominant clinical forms of leishmaniasis in Iran [7, 8].

Traditionally, for several decades the pentavalent antimonial drugs have been used as the first-line treatment and miltefosine, pentamidine and amphotericin B as the second-line treatment for CL [9]. However, all the available drugs are associated with challenges, including but not limited to lack of adherence to treatment, multiple injection regimens, low efficacy, high costs, therapeutic failure, potential emergence of resistance in parasite strains and significant side effects such as arthralgias, myalgias, leukopenia, pancreatitis, liver problems, cardiotoxicity and cardiac arrhythmia in the patients [9–12].

To date, no effective anti-leishmania vaccines have been approved, and considering the current challenges associated with anti-leishmanial drugs, the Tropical Disease Research (TDR) Program of the WHO has emphasized finding new anti-leishmanial therapeutic agents [9, 13]. Natural compounds, which are cost-effective and less toxic, are potential therapeutic candidates for the treatment of leishmaniasis [9].

In recent years, interest in the use of natural chemical compounds for the treatment of diseases has been growing [14]. In 2010, the WHO indicated that natural compounds can be exploited for the treatment of uncomplicated CL [15].

Recent evidence supports the use of natural insect products as drug candidates which can be exploited for their potential role in treating human diseases, an emerging field called “bugs as drugs” [16, 17].

There are reports on the successful treatment of chronic wounds using larval therapy (LT), especially *L. sericata* maggot therapy [18]. The ability of the larvae of *L. sericata* of the Calliphoridae family to remove dead tissue, stimulate tissue granulation and inhibit and eliminate wound biofilms has been widely investigated. Also, the larvae induce antimicrobial effects [19].

Several peptides with antimicrobial properties, including diptericin and defensins, have been isolated from larval excretion and secretion (ES) system of *L. sericata*. Also, serine proteases have been isolated from the *L. sericata* larval ES. These proteases inhibit DNA synthesis and disrupt the cell wall of microorganisms [19–21].

The anti-microbial properties of these peptides have been investigated in several studies against bacteria [22], fungi [23], viruses [24] and parasites [21]. About half of all anti-microbial peptides (AMPs) obtained from natural sources are from insects [16]. The potential of larval ES products in the treatment of CL has been investigated in in vitro and in vivo CL models [16, 21, 23, 25–27].

To the best of our knowledge, despite the promising potential of larval products of *L. sericata* described in the literature, the leishmanicidal effect of larval saliva and hemolymph of this fly against *L. major* is yet to be demonstrated. Also, a recent original article published by our group demonstrated that larval saliva and hemolymph of *L. sericata* have a therapeutic effect against the promastigote and amastigote forms of *L. tropica* [28].

In this context, the present study aimed at evaluating the leishmanicidal effect of *L. sericata* larval products against the extra- and intracellular forms of *L. major*. Moreover, the efficacy and safety of these products were evaluated in in vitro and in vivo experimental models: mammalian cells (murine macrophage cell line, J774A.1 cells and peritoneal macrophages) as in vitro model and *L. major*-infected mice as in vivo model. The therapeutic effects of the natural compounds were compared with the gold standard, glucantime.

## Methods

### Fly maintenance and rearing

In this study, larval products were prepared from laboratory strains of *L. sericata* at the third-instar stage. Live adult field strains of *L. sericata* were reared at the Cyclorrhapha Fly Insectary at the School of Public Health, Tehran University of Medical Sciences (TUMS), for 10 years. Adult *L. sericata* colonies were placed in 46 × 46 × 46-cm cloth cages in the insectarium under the following conditions: 27 ± 3 °C, 45 ± 5% RH and 16:8 h light:dark cycle (L:D).

The flies were maintained on a sugar diet (carbohydrate source) and were provided a piece of beef liver as a protein source and oviposition surface [29]. Batches of freshly laid eggs on the liver piece were transferred to a glass flask and maintained until larvae were hatched. The third-instar larvae were obtained and used for all subsequent experiments [30].

### Sterile larvae preparation

Third-instar larvae were collected from rearing glass flasks and washed in sterile distilled water. They were then placed in a 50-ml Falcon tube and disinfected by adding 4% Deconex for 3 min. The solution was then replaced with 70% isopropyl alcohol (IPA) with constant shaking for the same amount of time and then rinsed three times with sterile distilled water. Microbial infection of the samples was evaluated using laboratory tests before larval product preparation. Subsequently, larval salivary gland lysate (SGL) and hemolymph were prepared and used for further studies [28].

### Preparation of salivary gland lysate (SGL) of *Lucilia sericata*

Laboratory-reared third-instar larvae of *L. sericata* were subjected to salivary gland (SG) dissection. SGs were dissected under a stereo-microscope using fine forceps (Dumont #4) and needles (30 gauge) in cold fresh phosphate-buffered saline (PBS) with a pH of 7.2 and then transferred into 1.5-ml micro-tubes containing 40 μl fresh PBS in groups of 20 glands. Then, the salivary glands were stored at − 20 °C until further use.

The gland tissues were frozen in liquid nitrogen and thawed in boiling water (repeated for three cycles) just before use. Briefly, the salivary glands were placed in liquid nitrogen and thawed in boiling water for a few seconds and were then immediately placed back in the liquid nitrogen before thawing again in boiling water. This cycle was repeated for three cycles until there were only small breaks in the cell wall of the salivary glands, which allowed the contents of the glands to leak out. The homogenates were then centrifuged at 18,000 g for 15 min, and the supernatants were collected and used for subsequent experiments [31, 32].

### Preparation of larval hemolymph

To prepare hemolymph, 0.5-ml micro-tubes were used. A razor blade was used to cut the micro-tubes about 3–4 mm straight down the center. Small scissors were used to cut the frontal part of the laboratory-bred larvae (near the mouth hooks), and the cut parts were placed in the 0.5-ml micro-tubes in batches of ten larvae. The micro-tubes containing the specimens were placed in a larger microcentrifuge tube (1.5 ml), and the samples were centrifuged for 5–10 s to extract the hemolymph [28].

### Protein measurement

Protein concentration of the salivary gland lysate and hemolymph was quantified using BCA Protein Assay Kit (Takara Biotechnology, no. T9300A, Japan), according to the manufacturer’s instructions. Bovine serum albumin (BSA) was used as a standard protein for the assay in sodium azide.

### In vitro studies

#### Culture of *Leishmania major*

Specimens of the *Leishmania major* strain MRHO/IR/75/ER were isolated from an infected BALB/c mouse and maintained as promastigotes at 26 °C in Novy-Macneal-Nicolle (NNN) medium. The specimens were cultured in RPMI-1640 medium (Gibco, NM, USA) supplemented with 15% heat-inactivated fetal bovine serum (HIFBS), penicillin and streptomycin (100 ug/ml) with passages every 3 or 4 days. The bovine serum and antibiotics were obtained from Sigma (St. Louis, MO, USA). The mice were maintained in the animal house at the Center for Research and Training in Skin Disease and Leprosy (CRTSDL) of TUMS and were fed tap water and laboratory pellet chow.

#### Anti-promastigote assay (IC_50_)

Promastigotes of *L. major* were added to 96-well plates in RPMI1640 medium containing 15% FBS at a concentration of 1 × 10^5^ parasites per well. After 24 h, the promastigotes were treated with different concentrations of larval saliva and hemolymph (75, 150, 300, 450, 600 and 750 μg/ml) and subsequently incubated in RPMI medium without FBS at a temperature of 26 ± 1 °C for 24, 48, 72 and 96 h. *Leishmania major* specimens maintained in culture without treatment were used as a negative control, and glucantime was used as the reference clinical drug (positive control). The concentrations of glucantime used in this study were 25, 50, 100 and 250 μg/ml.

Following treatment, parasite viability (%) was evaluated using a 3-(4.5-dimethylthiazol-2-yl)-2, 5 diphenyltetrazolium bromide (MTT) assay. Briefly, 200 ul of MTT solution (0.5 mg/ml in PBS, prepared and filtered at the moment of use) was added to the parasite specimens and incubated for 4 h at 37 °C. The formazan crystals formed by the reduction of the MTT assay were solubilized by 100 μl dimethyl sulfoxide (DMSO), and the optical density (OD) of the plates was measured using the ELISA reader (Bio-Tek ELX 808 iu) at 560–630-nm wavelength [33]. Parasite viability (%) was determined at each concentration using the following formula:

Parasite viability = (average absorbance of quadruplicate treated wells)/(average absorbance of the control wells)*100.

The half-maximal IC_50_ was calculated by non-linear regression tests using GraphPad Prism^®^ 6.0.

#### Culture of the J774A.1 cell line

The murine cell line macrophages (Pasteur Institute, Tehran, Iran) were grown in DMEM medium under the following conditions; 37 °C, 5% CO_2_ and 95% humidity. The culture medium was supplemented with 15% FBS, penicillin and streptomycin (100 μg/ml). The viability percentage was determined using MTT colorimetric assay, and the CC_50_ was calculated [34].

#### Peritoneal macrophages from BALB/c mice

For this experiment, 4–5-week-old BALB/c mice were used. Peritoneal macrophages were isolated from the peritoneal cavity of the mice. Before cell extraction, sterile 3% thioglycollate broth was injected intraperitoneally (IP) into the peritoneal cavity to induce intraperitoneal inflammation.

The mice were anesthetized and killed using CO_2_, and each mouse was decontaminated by whole-body immersion in 70% alcohol or isopropanol. The specimens were then treated with 5 ml ice-cold PBS via intraperitoneal injection using a 5-ml syringe with a 25-G needle, and the abdomen was massaged for approximately 15–20 s. Subsequently, peritoneal fluid-containing cells were slowly aspirated from the peritoneal cavity of each mouse; approximately 4–4.5 ml of fluid was aspirated from each mouse. The aspirated fluid was dispensed in a centrifuge tube on ice, and the peritoneal cells were centrifuged for 10 min at 300 g.

Then, the cells were re-suspended in 1 ml DMEM medium supplemented with 10% FBS, and the cells were counted. The extracted peritoneal cells were incubated in a 5% CO_2_ incubator at 37 °C. Peritoneal macrophages adhered to the plastic surface and were ready for use in subsequent experiments [35].

#### Cytotoxicity in murine macrophages (CC_50_)

The pot-adherent peritoneal macrophages (1 × 10^5^ cells/well) and J774A.1 cells (5 × 10^4^ cells/well) were seeded in a 96-well culture plate and incubated overnight at 37 °C in 5% CO^2^. After removal of non-adherent cells, macrophages were treated with different concentrations of larval saliva and hemolymph (75, 150, 300, 450, 600 and 750 μg/ml) in DMEM medium under the same conditions for 24, 48, 72, 96 and 120 h. MTT colorimetric assay was used to quantify cell viability. Briefly, 200 ul MTT solution (MTT; SIGMA, St. Louis, MO, USA) was added per well (final concentration 0.5 mg/ml), and the plates were incubated for 4 h at 37 °C in 5% CO_2_. The formazan crystals were dissolved with DMSO (100 ul), and the absorbance was measured at 560–630 nm by a plate reader.

Cell viability was defined as the percentage of viability in the treated group compared to the control group and was calculated with the following formula: % (viable cells) = (average OD of samples treated with larval products/average OD of the untreated samples) × 100 [33].

Finally, the selectivity index (SI) of each larval product was calculated by dividing the CC_50_ value of the peritoneal macrophages of BALB/c mice and J774A.1 cells by the IC_50_ value of the promastigotes of *L. major* [36]. In this study, macrophage cells maintained in culture without treatment were used as a negative control, and glucantime was used at 25, 50, 100 and 250 μg/ml as the reference clinical drug (positive control).

#### Intracellular anti-amastigote activity

Peritoneal macrophages and J774A.1 cells were used as host cells for this experiment. The peritoneal macrophages (7 × 10^4^ cells/well) and J774A.1 cells (2 × 10^4^ cells/well) were seeded in an eight-well chamber slide for 24 h at 37 °C in 5% CO_2_. After allowing for adherence, the non-adherent cells were removed with RPMI medium at 37 °C. The host cells were then infected with a culture of stationary-phase promastigotes of *L. major* at an infection rate of 10:1 (parasite/macrophage) in the same medium supplemented with 10% FBS.

The plates were incubated for 24 h, and non-internalized parasites were washed and removed. The monolayers were treated with 150 and 450 μg/ml of larval saliva and hemolymph for 72 and 120 h under the same conditions. All experiments were performed in triplicate. Glucantime was used as a clinical standard (positive control), and infected macrophages with no treatment were used as a negative control.

Finally, the supernatant was removed, and the cells were studied microscopically. Each slide used for this experiment was washed with PBS, dried and fixed in methanol. Giemsa staining was applied for observation of the cells under the microscope. The anti-amastigote activity of the larval products was evaluated under a light microscope with immersion oil. The infection rates and number of amastigotes per 100 infected cells were determined by microscopy studies and compared with the controls (Table 1).

**Table 1:** Parameters for evaluating the anti-amastigote activity of larval-products

Parameter	Abbreviation	Equation
Infection percentage	I %	(Infected cells/100 randomly-chosen cells) × 100
Decreased in infection percentage	DI %	[(%I no treatment—%I treatment)/%I no treatment] × 100
Viability of amastigote percentage	V %	(amastigote treatment/amastigote no treatment) × 100
Decreased viability of amastigote percentage	DV %	[(amastigote no treatment—(amastigote treatment/no treatment] × 100
Parasite load	PL	amastigotes/infected cells
Survival index	SVI	%I × PL
Selectivity index	SI	CC_50_/IC_50_

### *In vivo *studies

#### Murine model of cutaneous leishmaniasis

Female and male BALB/c mice (age, 6–8 weeks), ranging from 20 to 25 g, were purchased from Pasteur Institute and maintained in CRTSDL’s animal facility. BALB/c mice (160 male and female) were inoculated subcutaneously (SC) in the left footpad with stationary-phase *L. major* (1*10^6^ in 50 ul volume) using a 30-gauge needle [37, 38].

Five weeks post-infection, the dimensions of the lesions (length and width) were measured using a digital caliper (Mitutoyo 500-196-30), and the mean values of the dimensions were determined. The infected mice were randomly distributed into eight treatment groups (8 male and 8 female groups) of ten mice each.

The animal models were treated with larval products and glucantime (positive control) via different routes of administration, as presented in Table 2. Animals in the larval product treatment groups were administered 37.5 mg/kg of saliva and hemolymph in a volume of 50 μl via intra-lesional (IL) route once a week and intra-peritoneal (IP) route every day for 5 weeks. The positive control group was treated by IP injection of glucantime (100–200 mg/kg) once a day for 5 weeks, and the negative control group remained untreated.

**Table 2:** Animal study: mice received larval products and glucantime via different routes of administration

No.	Groups name	Sex	Mice groups	Route of administration	Time-points
1	G1	M	Saliva-treated	IP	Daily
2	G2	M	Saliva-treated	IP	Weekly
3	G3	M	Saliva-treated	IL	Weekly
4	G1	F	Saliva-treated	IP	Daily
5	G2	F	Saliva-treated	IP	Weekly
6	G3	F	Saliva-treated	IL	Weekly
7	G4	M	Hemolymph-treated	IP	Daily
8	G5	M	Hemolymph-treated	IP	Weekly
9	G6	M	Hemolymph-treated	IL	Weekly
10	G4	F	Hemolymph-treated	IP	Daily
11	G5	F	Hemolymph-treated	IP	Weekly
12	G6	F	Hemolymph-treated	IL	Weekly
13	G7	M	Infected-untreated	IP	Daily
14	G7	F	Infected-untreated	IP	Daily
15	G8	M	Glucantime-treated	IP	Daily
16	G8	F	Glucantime-treated	IP	Daily

*F* female, *M* male, *IL* intra-lesional, *IP* intra-peritoneal

#### Safety test

Healthy male and female mice were injected intra-peritoneally with 37.5 mg/kg of saliva and hemolymph in a 50-μl volume every day for 15 days. Toxicity assessment was done by monitoring mouse weight changes and clinical signs of skin toxicity. All treated animals were monitored every day, and clinical signs such as skin irritation and laceration were recorded.

#### Evaluation of the effect of treatment on lesion development

The lesion size was evaluated weekly with a digital caliper (Mitutoyo 500-196-30), and the variation was determined. Average lesion development was determined as the difference in lesion size between left (infected) and right (uninfected) footpads. Three days after the end of the treatment, parasite quantification was evaluated in both draining inguinal lymph nodes and infected footpads by limiting dilution assay (LDA) [39, 40].

#### Quantitative parasite burden

The number of viable *L. major* parasites in the lymph nodes and infected footpads of the mice was determined by limiting dilution assay (LDA) in 96-well plates.

#### Limiting dilution assay (LDA)

The mice were killed by cervical dislocation, and tissues of the infected lymph nodes and hind footpads of three animals from each group were aseptically isolated, weighed and homogenized with a tissue grinder into 2 ml cold RPMI medium supplemented with 15% heat-inactivated FBS, penicillin and streptomycin (100 μg/ml) [39, 40]. The lesion size was measured in retained mice in each treated group at the 13th week.

Under sterile conditions, eight tenfold serial dilutions were prepared from the homogenates of lymph nodes and footpads of each mouse using RPMI medium. The diluted samples were then cultured in 96-well microtiter plates (Nunc AS, Roskilde, Denmark) containing a solid layer of rabbit blood agar. Samples were cultured in triplicate, and the plates were incubated at 25 ± 1 °C for 7–10 days.

Microplates were then observed for the presence or absence of viable promastigotes under an inverted microscope at 400× (Olympus, Tokyo, Japan). The number of viable parasites per tissue weight was obtained from the last dilution that contained parasites, using the following formula:

$$\left(-\log=\frac{\text{Parasite}\; \text{dilution}}{\text{Tissue}\; \text{weight}}\right)$$

### Statistical analysis

One-way ANOVA followed by Bonferroni multiple comparison tests was used to compare the differences in mean between the different treatment groups. The generalized estimation equation (GEE) method proposed by Liang and Zeger was used to determine the differences in the number of viable parasites and macrophage cells at different time points and under different treatments and concentrations. The GEE is an estimation method commonly used for marginal modeling of repeated data.

The CC_50_ and IC_50_ values of larval saliva and hemolymph against the host cells and promastigotes were estimated using GraphPad Prism (8.0.2). To investigate the susceptibility of amastigotes and promastigotes to the larval products and toxicity of the larval products on the macrophage cell types, infection rate (I%), decrease in infection rate (DI%), viability percentage of amastigotes (V%), percent decrease in viability of amastigotes (DV%), parasite load, survival index and selectivity index were estimated (Table 1). STATA version 13 MP was used for all the statistical analyses, and *p* values ≤ 0.05 were considered statistically significant.

## Results

### Protein concentration of larval products of *Lucilia sericata*

The average protein concentrations were estimated for laboratory-bred and field strains of *L. sericata* larvae. For the laboratory-bred parasites, the average protein concentration of the larval hemolymph and a pair of salivary glands was 314 μg and 14.7 μg, respectively. Also, for the field parasites, the average protein concentration for larval hemolymph and a pair of salivary glands was 213 μg and 5.7 μg, respectively.

### In vitro activity

#### Promastigotes assay

**Fig. 1:**
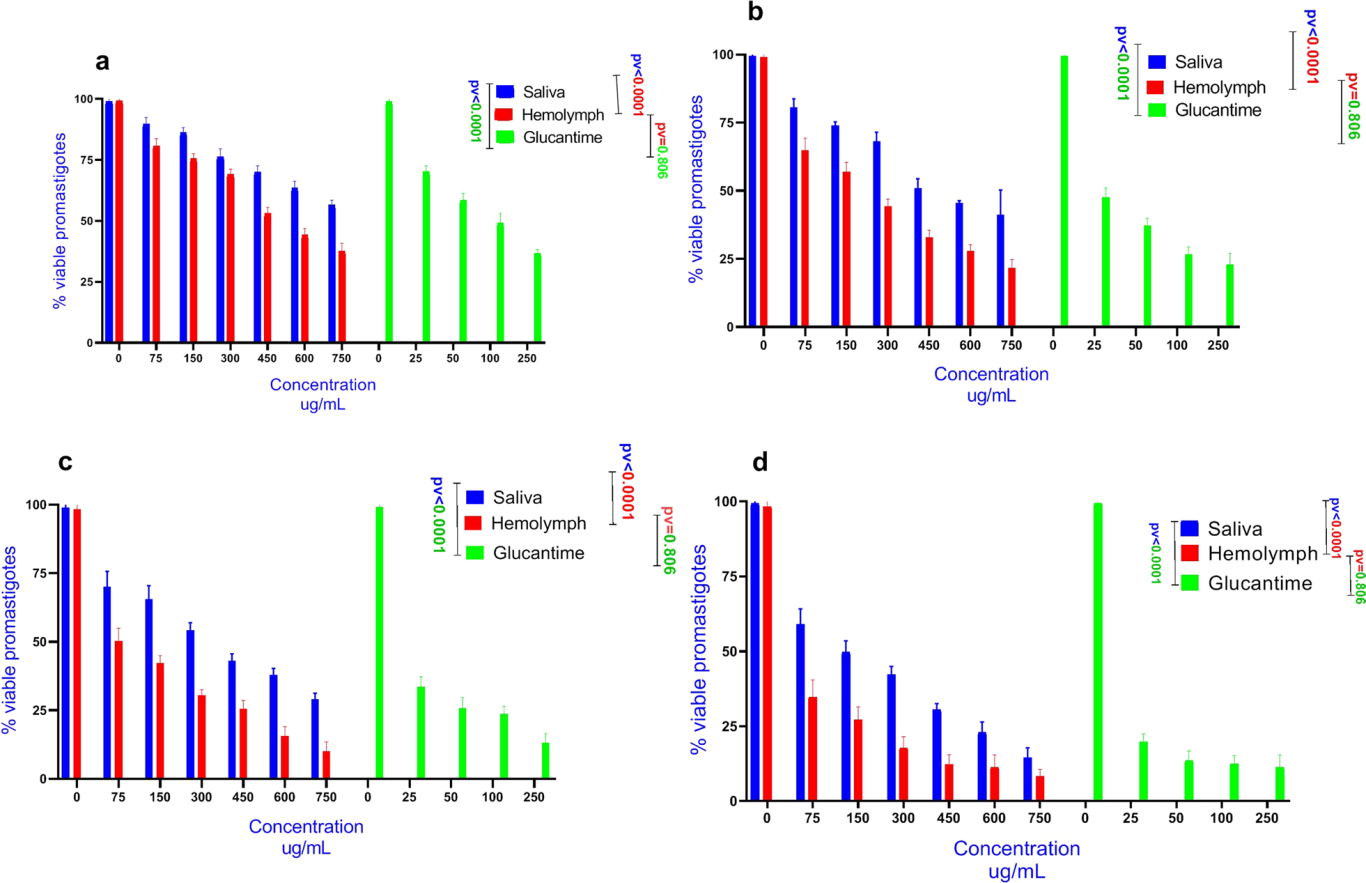
Viability percentage of *Leishmania major* promastigotes treated with different concentrations of *Lucilia sericata* larval saliva and hemolymph compared with glucantime. **a** %Viability of *L. major* promastigotes treated with different concentrations of larval products at 24 h. **b** %Viability of *L. major* promastigotes treated with larval products at 48 h. **c** %Viability of *L. major* promastigotes treated with larval products at 72 h. **d** %Viability of *L. major* promastigotes treated with larval products at 96 h

**Fig. 2:**
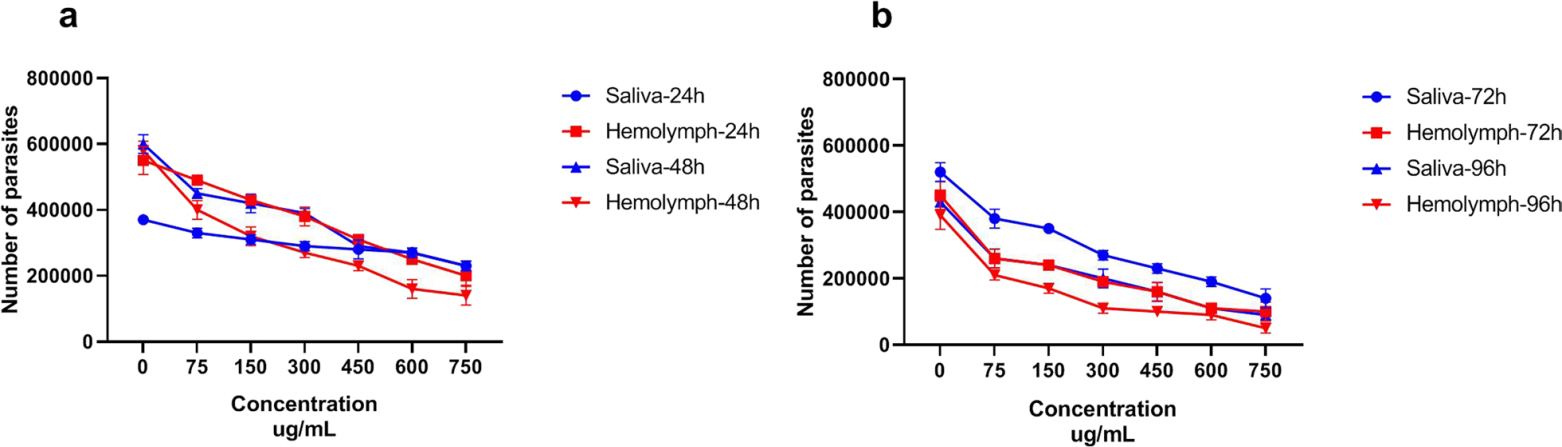
Number of viable *Leishmania major* promastigotes treated with *Lucilia sericata* larval products in different concentrations at different time points and counted in a Neubauer chamber. **a** Number of viable *L. major* at 24 and 48 h. **b** Number of viable *L. major* at 72 and 96 h

Larval saliva and hemolymph were potent inhibitors of parasite growth. The lowest viability percentage measured at 24 h was 55% and 38% for promastigotes treated with larval saliva and hemolymph, respectively. At 96 h exposure, the lowest viability percentage was 12% and 7% for treatment and hemolymph, respectively (Fig. 1). The number of live *L. major* promastigotes was obtained by direct observation under the light microscope, and the results are presented in Fig. 2.

**Fig. 3:**
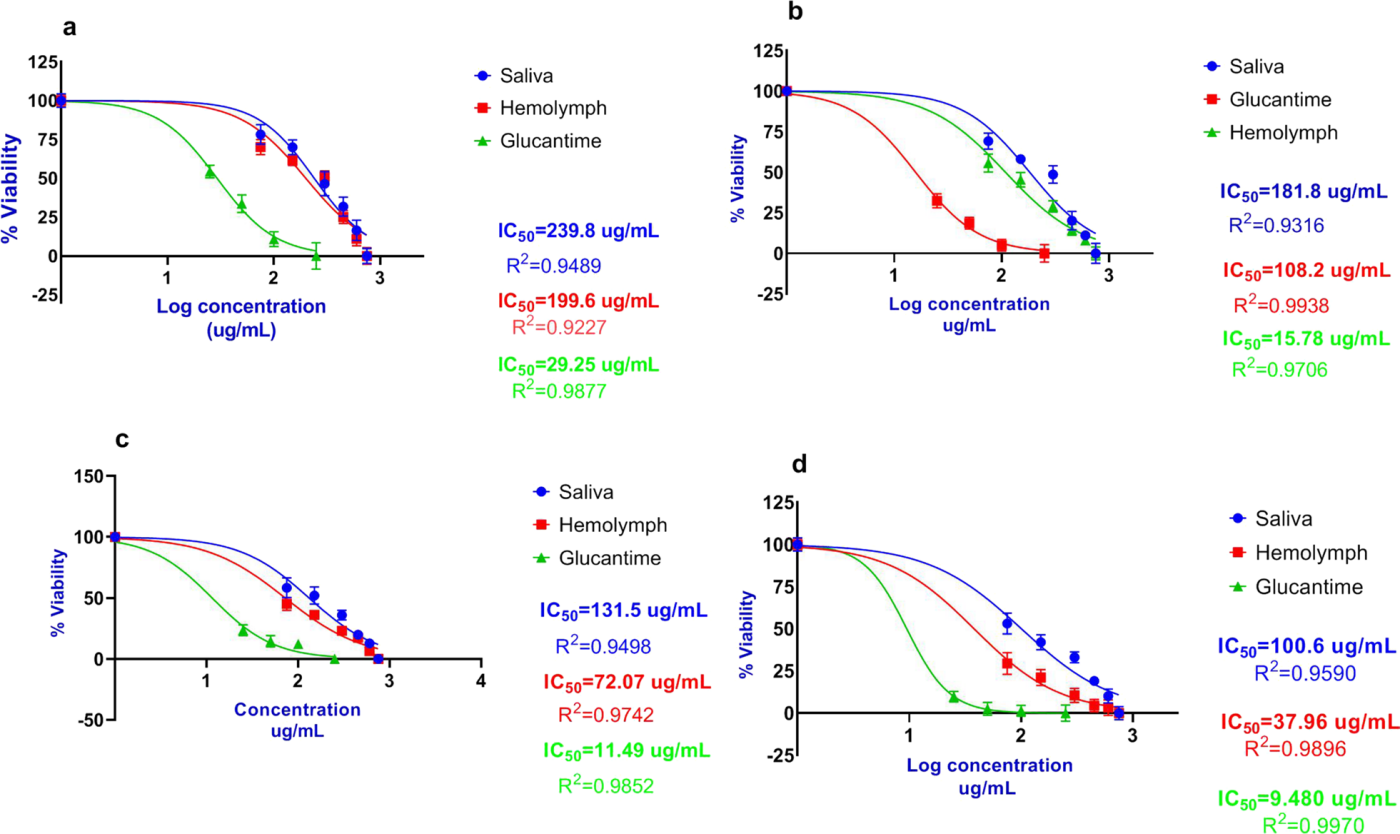
Dose-response curves regarding the effect of *Lucilia sericata* larval-derived products on *Leishmania major* promastigotes (IC_50_) using MTT assay. **a** Larval saliva IC_50_ vs. larval hemolymph IC_50_ at 24 h. **b** Larval saliva IC_50_ vs. larval hemolymph IC_50_ at 48 h. **c** Larval saliva IC_50_ vs. larval hemolymph IC_50_ at 72 h. **d** Larval saliva IC_50_ vs. larval hemolymph IC_50_ at 96 h and compared with standard treatment (glucantime)

Larval saliva displayed a significantly lower inhibitory activity than larval hemolymph (*t*_(54)_ = 5.05, *P* < *0.0001*) against *L. major* promastigotes. The lowest IC_50_ value was 100.6 μg/ml (log = 2.003) for larval saliva whereas it was 37.96 μg/ml (log = 1.579) for hemolymph at 96 h. Promastigote susceptibility to larval saliva and hemolymph was compared with glucantime tests and has been presented in Fig. 3.

The results of this study indicate that the viability of *L. major* promastigotes treated with both larval products (saliva and hemolymph) was significantly lower than for promastigotes in negative control at different concentrations (*F*_(2, 83)_ = 88.24, *P* = 0.001) and time points (*F*_(10, 83)_ = 310.83, *P* < 0.0001).

Also, a significantly higher number of viable promastigotes was observed when treated with saliva than with glucantime (*F*_(2, 83)_ = 101.28, *P* < 0.0001), but viability was not significantly different between promastigotes treated with hemolymph and glucantime (*F*_(2, 83)_ = 101.28, *P* = 0.806). In other words, the inhibition effect of larval hemolymph was greater than that of larval saliva (*t*_(54)_ = 5.05, *P* < *0.0001*) and similar to that of glucantime.

The results of this study indicate that there was a significant reduction in the viability of the parasites in a dose-dependent manner such that the inhibition effect of the larval products increased with an increase in concentration (*F*_(10, 83)_ = 530.50, *P* < *0.0001*) (Table 3). Also, there were no significant differences in the viability of promastigotes treated with larval products at concentrations 450, 600 and 750 μg/ml compared with glucantime at 25 (*F*_(10, 83)_ = 530.50, *P* = 0.476), 50 (*F*_(10, 83)_ = 530.50, *P* = 0.621) and 100 ug/ml (*F*_(10, 83)_ = 530.50, *P* = 0.819). Regarding the time of exposure, the viability percentage of *L. major* promastigotes diminished with an increase in time (*F*_(2, 83)_ = 101.28*, P* < 0.0001) (Table 4).

**Table 3:** Viability percentage of *Leishmania major* promastigotes in different concentrations of larval products

Time points (hours)	24 h				96 h		
Groups	Concentration (ug/ml)	Mean ± SD	Mean difference^a^ (95% CI) NC group	Statistical analysis^b^	Mean ± SD	Mean difference^a^ (95% CI) NC group	Statistical analysis^b^
Larval products	75	85.2 ± 7.7	− 13.8 (− 18.6 to − 9.1)	F_(10, 83)_ = 310.83 *P* < 0.0001*	46.8 ± 5.2	− 52.0 (− 57.8 to − 46.1)	F_(10, 83_) = 530.50 *P* < 0.0001
	150	80.9 ± 5.8	− 18.2 (− 22.9 to − 13.5)		38.5 ± 4.9	− 60.3 (− 66.2 to − 54.5)	
	300	72.6 ± 6.1	− 26.5 (− 31.2 to − 21.7)		30.0 ± 4.2	− 68.8 (− 74.7 to − 63.0)	
	450	61.5 ± 3.8	− 37.6 (− 42.3 to − 32.9)		21.3 ± 3.2	− 77.5 (− 83.3 to − 71.6)	
	600	53.8 ± 3.9	− 45.2 (− 49.9 to − 40.5)		17.1 ± 3.1	− 81.7 (− 87.6 to − 75.9)	
	750	47.0 ± 4.1	− 52.1 (− 56.8 to − 47.4)		11.3 ± 1.9	− 87.5 (− 93.3 to − 81.6)	
Glucantime	25	71.5 ± 6.1	− 27.6 (− 32.3 to − 22.9)	F_(10, 83)_ = 310.83 *P* < 0.0001	20.7 ± 2.6	− 78.1 (− 83.9 to − 72.2)	F_(10, 83)_ = 530.50 *P* < 0.0001
	50	59.6 ± 5.6	− 39.5 (− 44.2 to − 34.7)		14.0 ± 2.9	− 84.8 (− 90.7 to − 79.0)	
	100	48.3 ± 4.7	− 50.7 (− 55.4 to − 46.0)		11.8 ± 1.4	− 87.0 (− 92.8 to − 81.1)	
	250	39.0 ± 4.1	− 60.1 (− 64.8 to − 55.4)		10.3 ± 1.5	− 88.5 (− 94.3 to − 82.6)	
NC group	0	99.1 ± 6.8	Not applicable	Not applicable	98.8 ± 8.9	Not applicable	Not applicable

^a^Mean difference in treatment group compared to NC group

^b^Calculated based on two-way ANOVA

^*^Statistically significant < 0.05

*NC group* negative control or no treatment group

**Table 4:** Viability percentage of *Leishmania major* promastigotes in the different treatment groups at 24 and 96 h

Groups	Time points (hours)	Mean ± SD	Mean difference^a^ (95% CI)		*F*_(2, 83)_	*P* value^b^	
			NC group	PC group			
						NC group	PC group
Saliva	24 h	77.3 ± 3.8	− 21.8 (− 23.8 to − 19.7)	13.8 (11.8 to 15.7)	88.24	0.001^*^	0.001
	96 h	45.5 ± 3.4	− 53.5 (− 56.6 to − 51.4)	14.3 (11.9 to 16.9)	101.28	< 0.0001	< 0.0001
Hemolymph	24 h	65.6 ± 2.7	− 33.5 (− 36.1 to − 31.8)	2.0 (− 0.1 to 4.0)	88.24	0.001	0.806
	96 h	29.9 ± 2.2	− 68.9 (− 71.4 to − 65.1)	− 1.2 (− 3.6 to 1.2)	101.28	< 0.0001	0.455
Glucantime	24 h	63.5 ± 3.4	− 35.6 (− 37.6 to − 31.4)	Not applicable	88.24	0.001	Not applicable
	96 h	31.1 ± 2.9	− 67.7 (− 70.2 to − 64.8)	Not applicable	101.28	< 0.0001	Not applicable
NC Group	24 h	99.1 ± 6.8	Not applicable	35.6 (31.4 to 37.6)	88.24	Not applicable	0.001
	96 h	98.8 ± 8.9	Not applicable	67.7 (64.8 to 70.2)	101.28	Not applicable	< 0.0001

*NC group* negative control group or no treatment group, *PC group* positive control group (glucantime)

^a^Mean difference in larval products comparing NC and PC groups

^b^Calculated based on two-way ANOVA

^*^Statistically significant < 0.05

#### Larval product toxicity to macrophages cells

**Fig. 4:**
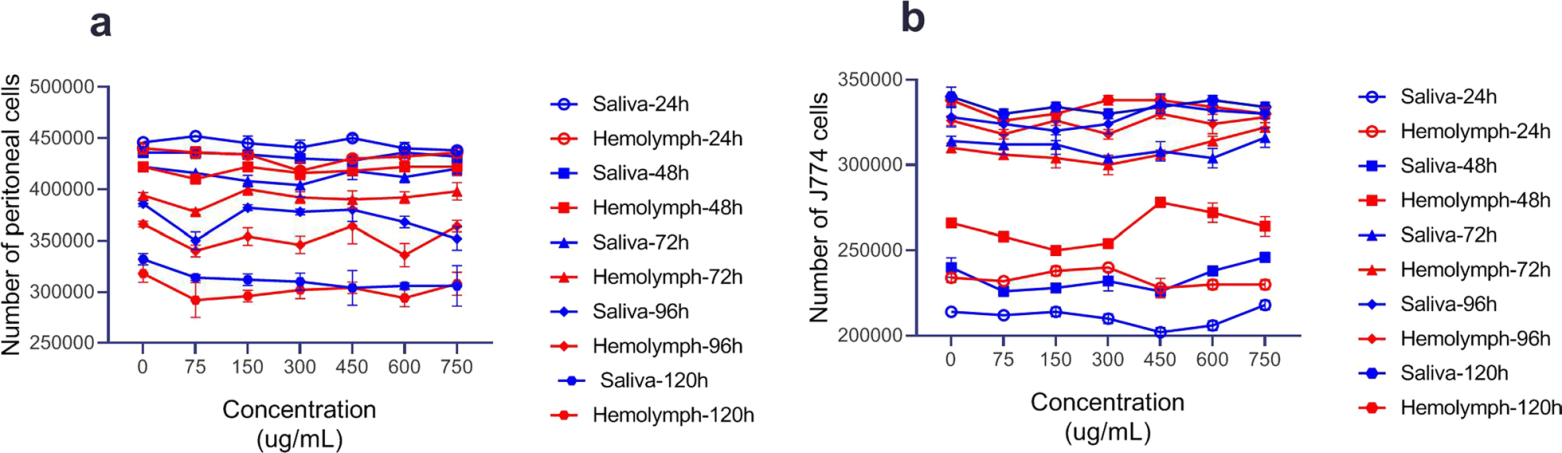
Number of viable cells treated with larval products by trypan blue in different concentrations at different time points and counted in a Neubauer chamber. **a** Number of peritoneal viable cells treated with larval products. **b** The number of J774A.1 viable cells treated with larval products

The cytotoxic effects of different concentrations of the larval products on both types of macrophages at 24, 48, 72, 96 and 120 h are presented in Fig. 4. The larval products had higher toxicity on *L. major* promastigotes, but they exhibited lower toxicity on macrophage cells at even higher concentrations after 120 h of exposure, with percent mortality of macrophage cells < 10%.

The number of live macrophage cells observed directly under the light microscope is presented in Fig. 4. MTT assay showed no significant difference in the viability percentage of the two types of macrophages when treated with larval saliva and glucantime (*t*_(34)_ = 0.57, *P* = 0.439), but a significant difference was observed between treatment with larval hemolymph and glucantime (*t*_(34)_ = 6.51, *P* = 0.007). The mean percent viability in the macrophages was 96.53 ± 3.57, 96.87 ± 3.44 and 97.40 ± 3.83 for treatment with hemolymph, saliva and glucantime, respectively.

The highest viability percentage was 97.98 ± 2.10 and 96.63 ± 2.90 for macrophages treated with larval saliva and hemolymph, respectively, compared with 98.93 ± 2.35 for glucantime treatment. Also, the lowest viability in the macrophages treated with saliva and hemolymph were 93.98 ± 5.15 and 93.93 ± 5.22, respectively, compared with 94.41 ± 6.17 for treatment with glucantime. Moreover, the cell viability percentage was significantly different when each macrophage type (*t*_(90)_ = 21.42, *P* < 0.0001) was studied under all three types of treatments. The viability percentages were 96.86 ± 3.15 and 93.73 ± 5.60 for J774 and peritoneal cells, respectively.

**Fig. 5:**
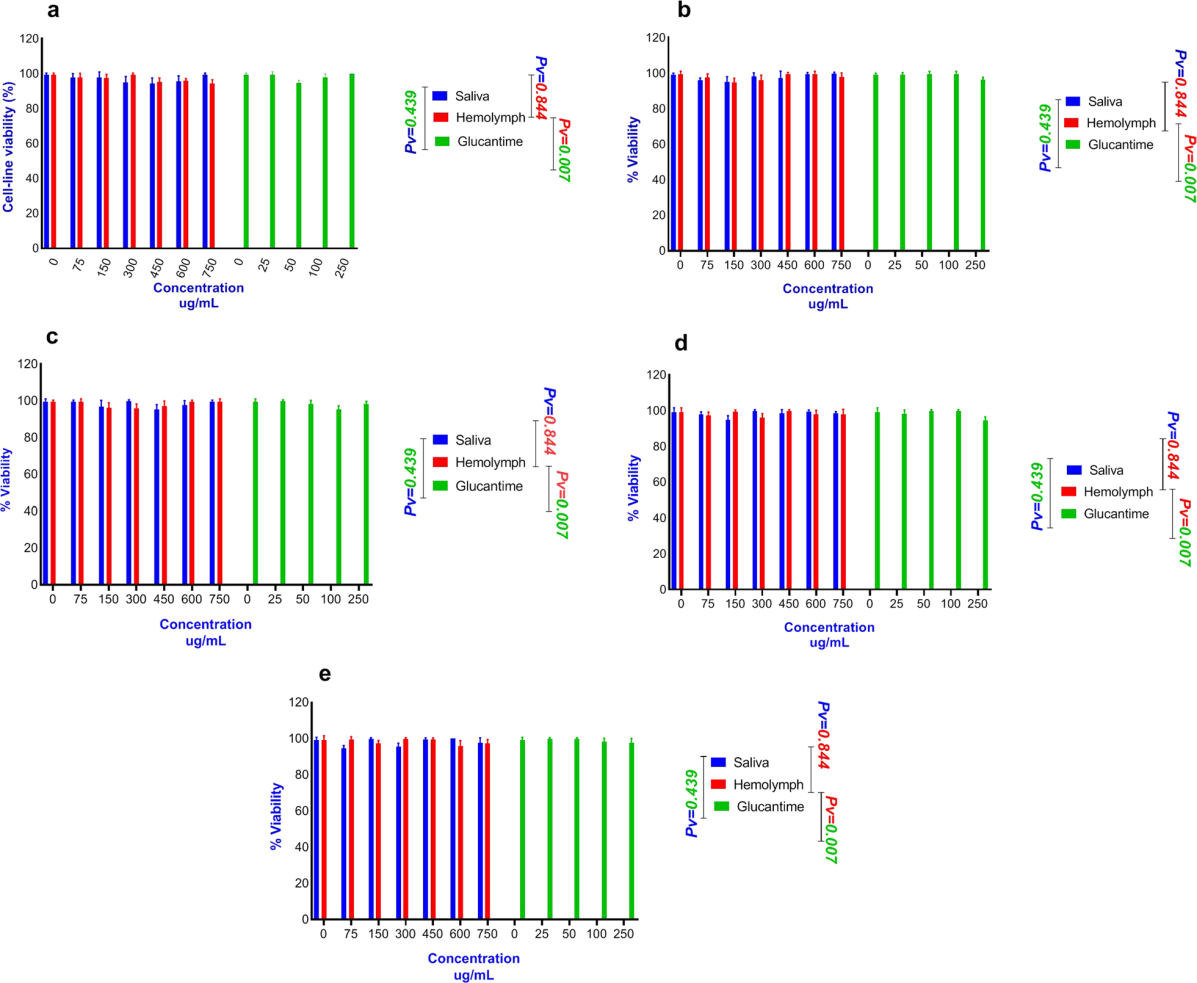
Cytotoxicity test of different concentrations of *Lucilia sericata* larval products on J774A.1 murine macrophage cell line at different time points compared with glucantime. **a** Cell viability in cells treated with larval products at 24 h. **b** Cell viability in cells treated with larval products at 48 h. **c** Cell viability in cells treated with larval products at 72 h. **d** Cell viability in cells treated with larval products at 96 h. **e** Cell viability in cells treated with larval products at 120 h

**Fig. 6:**
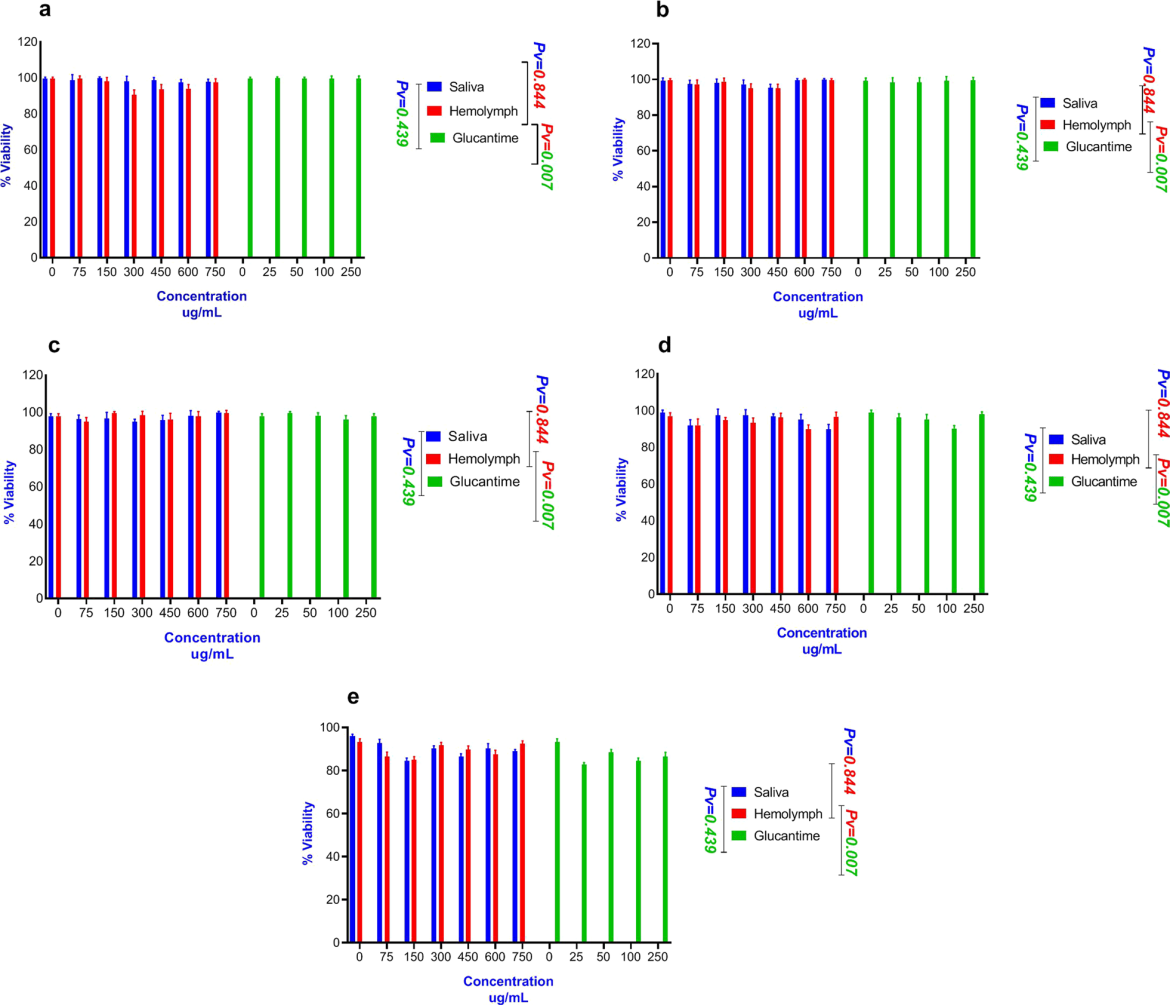
Cytotoxicity test of the effects of different concentrations of *Lucilia sericata* larval products on peritoneal macrophage cell line at different time points compared with glucantime. **a** Cell viability in cells treated with larval products at 24 h. **b** Cell viability in cells treated with larval products at 48 h. **c** Cell viability in cells treated with larval products at 72 h. **d** Cell viability in cells treated with larval products at 96 h. **e** Cell viability treated with larval products at 120 h

The results of the MTT assay of larval products on both macrophage types were determined at different time points after incubation, and the results were compared with the control groups. As shown in Figs. 4, 5 and 6, larval products did not affect the cell viability of the two types of macrophage at different time points, but the number of peritoneal cells decreased after day 5, which may be because peritoneal cells cannot multiply compared to J774 cells. In other words, a decrease in the percent viability of peritoneal cells does not indicate an increased sensitivity to the natural compounds compared to J774 cells or greater resistance of J774 compared to peritoneal cells. This could mainly be due to the non-proliferation activity of peritoneal cells.

The selectivity indexes (SI) of *L. sericata* larval saliva and hemolymph are shown in Table 5. The selectivity index of *L. sericata* larval hemolymph was 15.75, suggesting a potent anti-Leishmanial compound.

**Table 5:** Selectivity index for *Leishmania major* promastigotes treatment with saliva and hemolymph at different time points

Selectivity index (CC_50_/IC_50_)				
Time Treatment	24 h	48 h	72 h	96 h
Saliva Hemolymph	3.12 3.76	4.12 5.93	5.70 10.31	7.45 15.75

#### Intracellular anti-amastigote activity

**Fig. 7:**
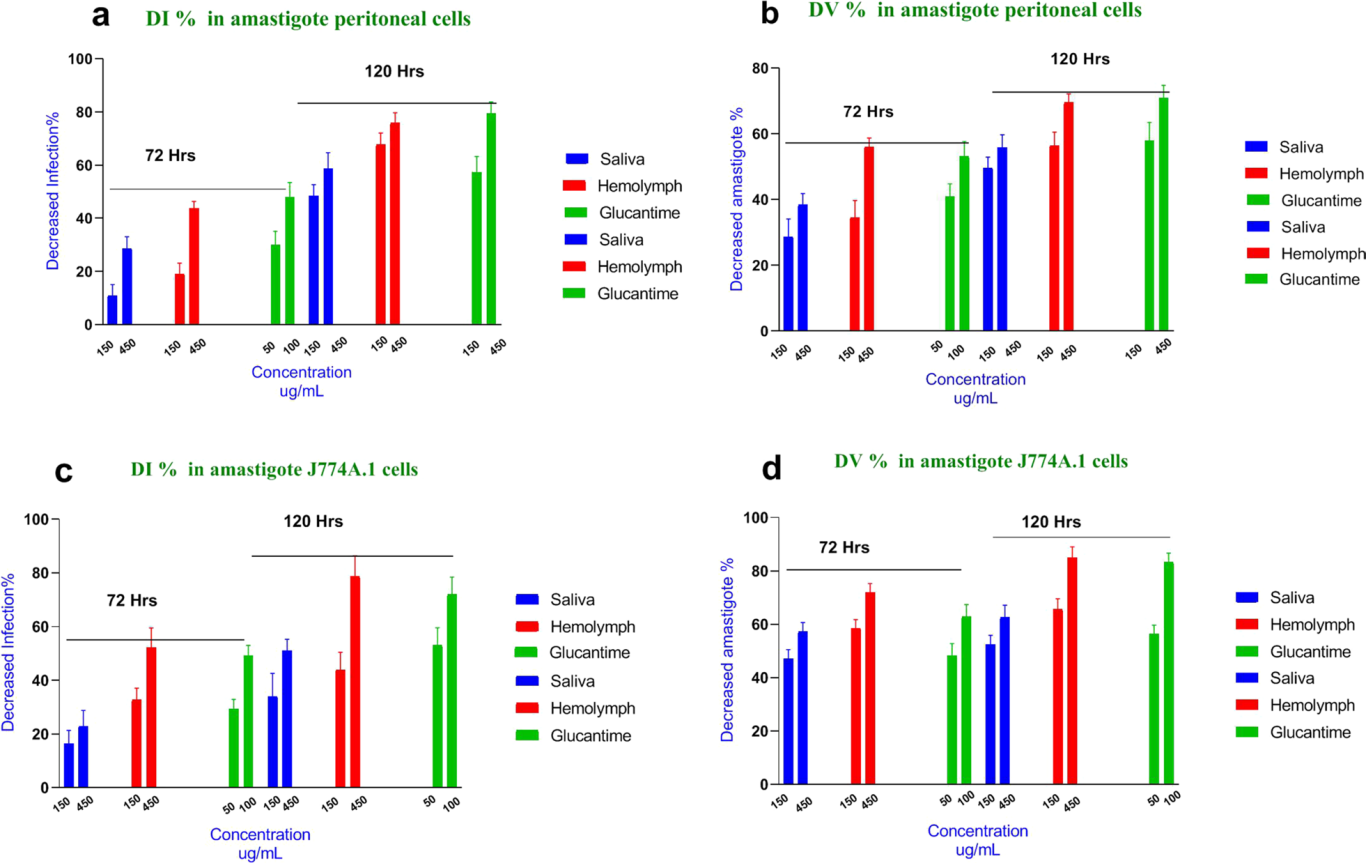
In vitro larval products’ effect on both macrophage types infected with *Leishmania major* at 72 and 120 h compared with glucantime. **a** Decrease in infection percentage (DI%) in *L. major* amastigote peritoneal cells. **b** Decrease in amastigote viability (DV%) in *L. major* amastigote peritoneal cells. **c** Decrease in infection percentage (DI%) in *L. major* amastigote J774A.1 cells. **d** Decrease in amastigote viability (DV%) in *L. major* amastigote J774A.1 cells

The infection rate (I%), decrease in infection rate (DI%), viability percentage of amastigotes (V%) and percent decrease in amastigote viability (DV%) for treatments with saliva and hemolymph were estimated, and the results were compared with glucantime. The findings are summarized in Table 6 and Fig. 7. A significant reduction was observed in I% (*F*_(2, 105)_ = 2.20, *P* = 0.001) and V% (*F*_(2, 105)_ = 3.91, *P* < 0.0001) 72 and 120 h incubation with larval products compared with the negative control group.

**Table 6:** Effect of *Lucilia sericata* larval products on *Leishmania major* amastigotes in in vitro conditions

Treatment	Dosages (ug/mL)			Amastigote *L. major*/peritoneal cell		Amastigote *L. major*/J774 cell-line			
Time(h)	I%			V%		I %		V%	
	72 h		120 h	72 h	120 h	72 h	120 h	72 h	120 h
No-treatment control	0	73.7 ± 3.7	68.3 ± 4.1	100 ± 0	100 ± 0	61.3 ± 3.7	47 ± 2.9	100 ± 0	100 ± 0
Saliva	150	65.7 ± 5	35 ± 3.5	71.2 ± 4	50.5 ± 4.3	51 ± 3.9	31 ± 2.1	52.8 ± 4.2	47.5 ± 3.3
	450	52.7 ± 3.6	28 ± 2.8	61.5 ± 3.7	44.2 ± 3.1	41 ± 1.8	23.3 ± 2.5	42.7 ± 1.4	37.3 ± 3.7
Hemolymph	150	59 ± 2.5	22.7 ± 1.8	65.7 ± 4.2	43.6 ± 2.9	47 ± 4.7	26.3 ± 3.6	41.4 ± 3.5	34.3 ± 4
	450	41.7 ± 3.7	16.3 ± 4.4	43.7 ± 3.3	30.5 ± 1.8	29.3 ± 321	10 ± 1.1	31.7 ± 2.8	14.8 ± 2.7
Positive control Glucantime	50	51 ± 2.3	29.3 ± 3.8	59.2 ± 3.7	41 ± 1.8	43.8 ± 1.8	22 ± 1.5	51.7 ± 2.5	43.5 ± 1.4
	100	38.3 ± 3.1	14 ± 2.16	46.2 ± 2.7	29 ± 2.8	31.7 ± 2.6	12.5 ± 1.2	37.1 ± 4	16.7 ± 1.8

*I%* infection percentage*, V%* viability of amastigote percentage

The results of the present study show a significant difference in the infection rate of *L. major* amastigotes between treatment with larval saliva and glucantime (*t*_(70)_ = 1.860, *P* = 0.051), but there was no significant difference when treatment with hemolymph was compared with glucantime (*t*_(70)_ = 0.91, *P* = 0.880). Furthermore, for the same tested concentrations, larval saliva (*t*_(70)_ = 0.88, *P* = *0.392*) and hemolymph (*t*_(70)_ = 0.37, *P* = *0.923*) were able to significantly reduce the number of amastigotes per macrophage (V%) compared to glucantime (Table 6).

In general, the infection rate decreased with an increase in the concentration of larval products (*t*_(2)_ = 34.72, *P* < *0.0001*) and time point (*t*_(35)_ = 9.47, *P* = 0.001). Also, at higher concentrations (*t*_(2)_ = 31.37, *P* < *0.0001*) and time points (*t*_(35)_ = 6.70, *P* = 0.003), larval products were able to significantly decrease the number of amastigotes per macrophage (Table 6). As shown in Fig. 7, the highest DI% and DV% were 58.82 and 62.69% for intracellular amastigotes treated with larval saliva and 78.72 and 69.51% for intracellular amastigotes treated with larval hemolymph, respectively. We observed an increase in DI% (decreased in infection percentage) and DV% (decrease in amastigote viability percentage) when infected macrophages were treated for a longer time with higher concentrations of larval saliva and hemolymph (Fig. 7). An ideal drug candidate should have a lower I% and V and a higher DI% and DV%, which is similar to the properties of larval saliva and hemolymph observed in this study.

There were no statistically significant differences in I% and V% between treatment with larval products at 150 μg/ml and glucantime at 50 μg/ml (*t*_(34)_ = 1.34, *P* = 0.321) and (*t*_(34)_ = 1.19, *P* = 0.996), respectively. Also, the same result was observed in treatment with larval products at 450 μg/ml and glucantime at 100 μg/ml (*t*_(34)_ = 1.45, *P* = *0.408*) and (*t*_(34)_ = 1.62, *P* = *0.320*), respectively (Table 4). Finally, the intracellular anti-amastigote activity was similar between 150 ug/mg hemolymph and 50 ug/mg glucantime (*t*_(22)_ = 1.07, *P* = 0.880) (Table 6). The results of average survival index (SVI) and parasite load (PL) are presented in Tables 7 and 8.

**Table 7:** Parameters for evaluating the anti-amastigote activity of larval products on peritoneal macrophages infected with *Leishmania major*

Treatment	Dosages (ug/mL)	PL		SVI	
		72 Hrs	120Hrs	72 Hrs	120 Hrs
No-treatment control	0	2.34 ± 0.65	2.97 ± 0.34	172.45 ± 9.3	201.8 ± 8.1
Saliva	150	2.07 ± 0.61	1.99 ± 0.48	135.3 ± 8.8	69.65 ± 6.9
	450	2.01 ± 0.47	1.81 ± 0.66	105.9 ± 6.9	50.68 ± 5.3
Hemolymph	150	2.00 ± 0.80	1.76 ± 0.42	118.6 ± 5.1	59.40 ± 7.7
	450	1.85 ± 0.25	1.35 ± 0.57	77.19 ± 7.8	47.73 ± 4.4
Positive control-Glucantime	50	1.91 ± 0.48	1.41 ± 0.48	97.07 ± 5.7	41.04 ± 5.5
	100	1.79 ± 0.36	1.33 ± 0.39	68.48 ± 3.5	21.82 ± 3.1

*PL* parasite load, *SVI* survival index

**Table 8:** Parameters for evaluating the anti-amastigote activity of larval products on J774A.1 macrophages infected with *Leishmania major*

Treatment	Dosages (ug/ml)	PL		SVI	
		120 h	72 h	120 h	72 h
No treatment control	0	2.86 ± 0.89	2.05 ± 0.71	175.3 ± 9.3	97.8 ± 8.1
Saliva	150	2.09 ± 0.58	1.98 ± 0.69	106.6 ± 7.81	61.38 ± 5.9
	450	1.90 ± 0.33	1.81 ± 0.43	78.00 ± 6.8	42.29 ± 4.3
Hemolymph	150	1.85 ± 0.53	1.77 ± 0.60	87.00 ± 8.1	47.16 ± 6.7
	450	1.76 ± 0.42	1.69 ± 0.32	52.47 ± 3.8	17.74 ± 2.4
Positive control glucantime	50	1.92 ± 0.51	1.72 ± 0.38	84.21 ± 5.7	38.85 ± 4.5
	100	1.69 ± 0.34	1.45 ± 0.31	53.78 ± 3.5	18.82 ± 1.1

*PL* parasite load, *SVI* survival index

### In vivo evaluation

#### Lesion size

**Fig. 8:**
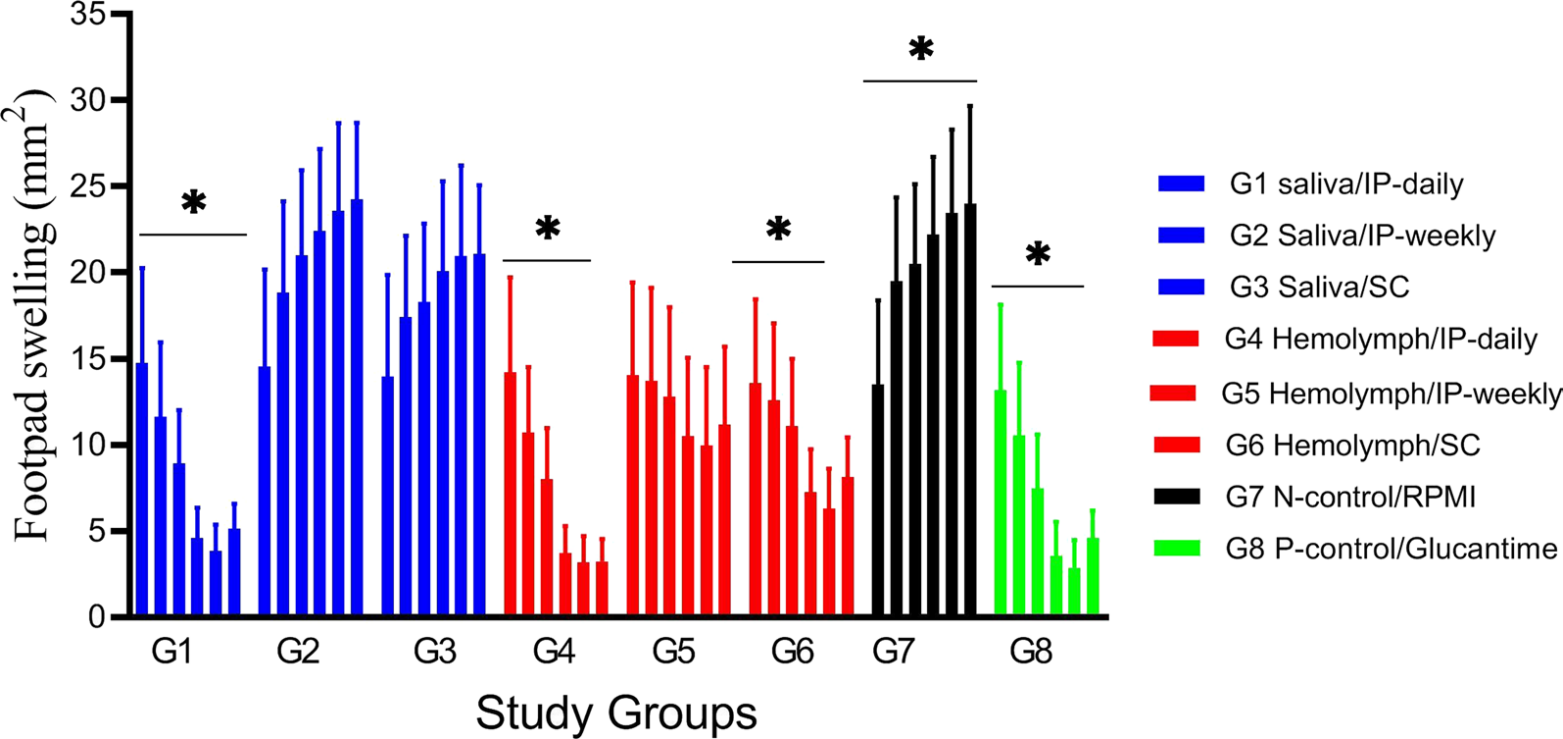
Progress of lesion size in inoculated BALB/c mice by *Leishmania major* in study groups at 6 weeks. The bar represents the means ± standard errors of means. There was a significant difference (*P* < 0.05) between the larval product-treated groups (G1, G4, and G6) and the untreated group of mice (G7)

Lesion size was evaluated weekly after *L. major* inoculation. Mice in G4 and G2 groups had the smallest and largest mean lesion size in the 5th week of treatment, respectively. Also, the results demonstrate that 4 weeks (week 13) after the end of the treatment period, lesion dimensions increased in all experimental and positive control groups except the G4 group (Fig. 8).

**Fig. 9:**
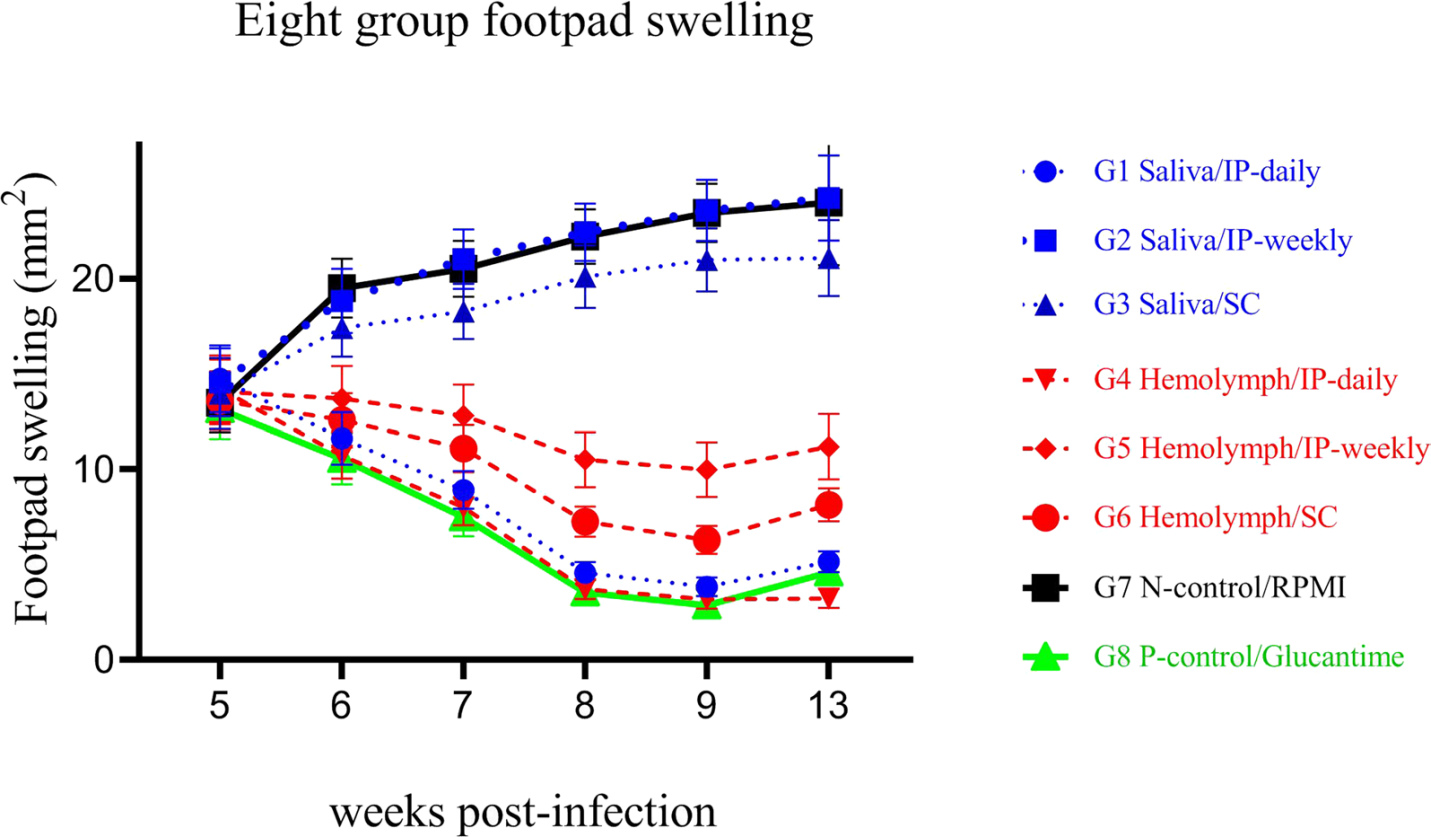
Footpad swelling in BALB/c mice treated with larval products and glucantime (G8) with different routes of administration at 6 weeks post-infection compared to the negative control group (G7)

The lesion development in BALB/c mice over 6 weeks post-infection in the study groups is presented in Fig. 9. Lesion size after the end of the treatment period in the G1, G4 and G6 groups was significantly (*F*_(7, 38)_ = 8.54, *P* < 0.0001) smaller than in the other groups (G2, G3, and G5) and the untreated group (G7) (Table 9).

**Table 9:** Comparison of the lesion size (mm²) before, immediately after and 4 weeks after the intervention in treatment groups

Study group	Mean ± SD			Mean difference^a^ (95% CI) G7 group^a^	Statistical analysis^b^
	Before intervention (week 5)	Immediately after intervention (week 9)	4 weeks after the intervention (week 13)		
G1	14.7 ± 2.5	3.8 ± 1.2	5.5 ± 1.3	− 19.1 (− 25.6 to − 12.5)	*F*_(7, 38) =_8.54, *P* = 0.001^*^
G2	14.5 ± 2.6	23.5 ± 4.0	23.9 ± 4.2	0.63 (− 3.9 to 2.5)	*F*_(7, 38) =_8.54, *P* = 0.811
G3	13.9 ± 2.8	20.9 ± 2.5	20.9 ± 2.7	− 1.9 (− 4.5 to 3.5)	*F*_(7, 38) =_8.54, *P* = 0.811
G4	14.2 ± 2.5	3.1 ± 1.1	3.0 ± 1.2	− 19.7(− 26.3 to − 13.1)	*F*_(7, 38) =_8.54, *P* = 0.001
G5	14.0 ± 2.3	9.9 ± 2.1	10.9 ± 2.3	− 12.9 (− 15.5 to 2.3)	*F*_(7, 38) =_8.54, *P* = 0.811
G6	14.1 ± 2.9	8.2 ± 1.8	7.7 ± 1.9	− 14.6 (− 19.6 to − 10.5)	*F*_(7, 38) =_8.54, *P* = 0.001
G7	12.8 ± 2.6	22.9 ± 2.8	24.0 ± 4.4	Not applicable	Not applicable
G8	13.1 ± 2.3	11.7 ± 1.9	4.7 ± 1.4	− 20.0 (− 24.6 to − 17.5)	*F*_(7, 38) =_8.54, *P* = 0.001

^a^Mean difference treatment group compared to G7 (no treatment group) at week 9

^b^Calculated based on ANOVA-ANCOVA

^*^Statistically significant < 0.05

*G7* negative control or no treatment group

The footpad thickness was measured for 6 weeks. However, there were no significant differences in the mean lesion size in groups G2, G3 and G5 compared to the negative control group (G7) (*F*_(7, 38)_ = 8.54, *P* = 0.811) (Table 9) and in G1, G4 and G6 groups compared with the glucantime group (G8) (*F*_(7, 38)_ = 8.54, *P* = 1.000). Finally, no significant sex differences were observed in the mean size of lesion in all treatment groups (*F*_(1, 5) =_1.10, *P* = 0.935). Thus, our findings suggest that response to treatment with larval saliva and hemolymph was not sex-dependent.

#### Parasite burden

**Fig. 10:**
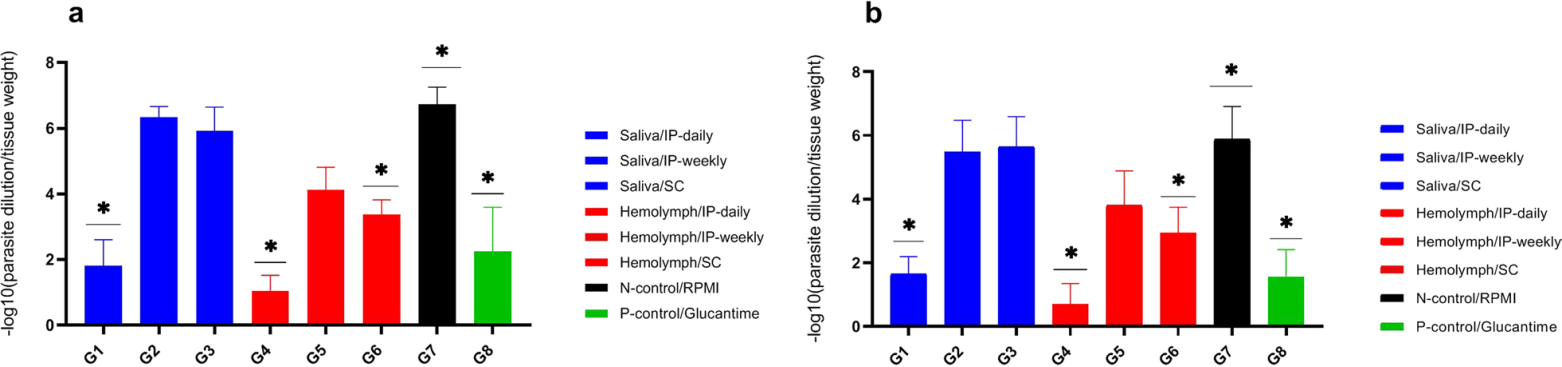
Footpad and lymph node parasite burden in *Leishmania major*-infected BALB/c mice treated with larval saliva and hemolymph. The number of viable parasites per tissue was determined by the following formula: —log10 (parasite dilution/tissue weight). The bar represents the means ± standard errors of the means. There was a significant difference (*P* < 0.05) between the larval product-treated groups (G1, G4 and G6) and an untreated group of mice (G7). **a** Parasite burden in the foot; **b** parasite burden in lymph nodes

The number of viable *L. major* parasites was determined in the lymph nodes and infected footpads of different treatment groups of mice at day 3 post-treatment (Fig. 10a, b). The parasite burden in the footpads and lymph nodes in G1, G4 and G6 mice was significantly lower (*F*_(7, 16)_ = 66.39, *P* < 0.0001) than in the untreated control group (G7) and similar *F*_(7, 16)_ = 66.39, *P* = 0.931; (*F*_(7, 16)_ = 66.39, *P* = 0.126; *F*_(7, 16)_ = 66.39, *P* = 0.113, respectively, to those treated with glucantime (G8).

The mean reductions in parasite burden in the footpads and lymph nodes in G1, G4 and G6 mice were 1.81 ± 0.74 and 1.65 ± 0.50 (G1), 1.03 ± 0.45 and 0.69 ± 0.24 (G4) and 3.37 ± 0.88 and 2.94 ± 0.78 (G6), respectively, compared to glucantime with a parasite burden of 2.24 ± 1.26 in the footpads and 1.54 ± 0.81 in the lymph nodes. Also, there was no significant difference among the G2, G3 and G5 groups compared with the untreated control group (G7) (*F*_(7, 16)_ = 66.39, *P* = 0.593). The parasite burden in the footpads and lymph nodes in G2, G3 and G5 mice were 6.33 ± 0.31 and 5.48 ± 0.94 (G2), 5.92 ± 0.68 and 5.65 ± 0.88 (G3) and 4.12 ± 0.64 and 3.81 ± 1.01 (G5), respectively, compared to the untreated control group with a parasite burden of 6.72 ± 0.49 in the footpads and 5.89 ± 0.96 in the lymph nodes (Table 10).

**Table 10:** Comparison of the parasite burden 3 days after the intervention in treatment groups

Study group	Parasite burden in footpads mean ± SD	Mean difference^a^ (95% CI) G7 group	Statistical analysis	Parasite burden in LN mean ± SD	Mean difference^a^ (95% CI) G7 group	Statistical analysis^b^
G1	1.8 ± 0.2	− 4.9 (− 5.6 to -3.4)	*F*_(7, 16) =_66.39, *P* < 0.0001*	1.6 ± 0.2	− 4.2 (− 5.5 to − 2.8)	*F*_(7, 16) =_51.24, *P* = 0.001^c^
G2	6.3 ± 1.1	− 0.3 (− 1.5 to 0.3)	*F*_(7, 16) =_66.39, *P* = 0.593	5.4 ± 0.9	− 0.4 (− 1.7 to 0.9)	*F*_(7, 16) =_51.24, *P* = 0.954
G3	5.9 ± 0.9	− 0.8 (− 1.9 to -12.5)	*F*_(7, 16) =_66.39, *P* = 0.593	5.6 ± 1.1	− 0.2 (− 1.5 to 1.1)	*F*_(7, 16) =_51.24, *P* = 0.954
G4	1.0 ± 0.5	− 5.7 (− 6.8 to − 4.5)	*F*_(7, 16) =_66.39, *P* < 0.0001	0.7 ± 0.1	− 5.2 (− 6.5 to − 3.8)	*F*_(7, 16) =_51.24, *P* = 0.001
G5	4.1 ± 0.5	− 1.6 (− 2.7 to 1.2)	*F*_(7, 16) =_66.39, *P* = 0.593	3.8 ± 0.9	− 1.2 (− 2.4 to 1.8)	*F*_(7, 16) =_51.24, *P* = 0.954
G6	3.3 ± 0.3	− 3.3 (− 4.4 to − 2.2)	*F*_(7, 16) =_66.39, *P* < 0.0001	2.9 ± 0.6	− 2.9 (− 4.2 to − 1.5)	*F*_(7, 16) =_51.24, *P* = 0.001
G7	6.7 ± 0.8	Not applicable	–	5.8 ± 1.2	Not applicable	–
G8	2.2 ± 0.4	− 4.4 (− 5.5 to − 3.3)	*F*_(7, 16) =_66.39, *P* < 0.0001	1.5 ± 0.3	− 4.3 (− 5.6 to − 2.9)	*F*_(7, 16) =_51.24, *P* = 0.001

^**a**^Mean difference in treatment group compared to NC group

^b^Calculated based on one-way ANOVA

^*^Statistically significant at < 0.05

*G7* negative control or no treatment group

## Discussion

Over the last decade, considerable attention has been given to *L*. *sericata* larval-derived natural products in an attempt to discover novel leishmanicidal compounds [16]. To the best of our knowledge, no study, except for our previous study, has examined the effects of larval saliva and hemolymph on *Leishmania* promastigotes [28]. Given the challenges associated with the current anti-leishmaniasis treatment, this study presents* in vitro* anti-leishmania activity and in vivo efficacy of larval products from *L*. *sericata* against *L*. *major*, a widely distributed species in Iran that causes diffuse CL [7].

The leishmanicidal activity of larval excretion/secretion (ES) products against *Leishmania* species has been demonstrated in several previous studies [20, 21, 26, 41]. However, to the best of our knowledge, no study has investigated the possible anti-microbial role of larval saliva and hemolymph of *L. sericata* against the clinical forms of *L*. *major*.

The results of the present study confirm the in vitro inhibitory effects of larval saliva and hemolymph against *L*. *major* proliferation. Herein, our results demonstrate that larval saliva and hemolymph have a leishmanicidal effect on promastigote forms of *L. major*. Hemolymph, associated with a lower host cell viability percentage, was highly toxic to promastigote forms of *L. major* compared with saliva. In the present study, the inhibitory effect of the larval products increased with an increase in concentration and time of exposure.

Our study is the first to determine the IC_50_ values of larval saliva and hemolymph (100.6–37.96 μg/ml, respectively) against *L. major* at lower inhibitory concentration compared with that of *L. tropica* (IC_50_ 134 and 60.44 μg/ml, respectively) [28]. The higher inhibitory effect of larval products on *L. major* compared with *L. tropica* is probably due to the influence of genetic and biological differences [42].

The present study revealed that larval saliva and hemolymph showed more potent cytotoxicity against promastigote forms and very low cytotoxic effects on peritoneal macrophages and J774A.1 cells. In other words, treatment with higher concentrations of larval products had greater effects on promastigotes than macrophage cells, with a percentage of mortality in macrophage cells < 10% even at higher concentrations.

The viability percentage of both types of macrophages was significantly different between treatment with larval hemolymph and glucantime. Also, no significant difference in the percent viability of both macrophage types was observed when treatments with larval saliva and glucantime were compared. In this regard, the MTT data suggest that saliva presents lower toxicity to macrophage cells than hemolymph.

The experimental SI in this study was > 10, indicating that the larval products are relatively safe leishmanicidal agents. However, it seems that the cytotoxic effects of larval products and glucantime were slightly higher in the peritoneal macrophage cells compared with the J774A.1 cell line, which may be due to the influence of genetic manipulations of the cell line [43] and the non-proliferative behavior of peritoneal cells. Therefore, the reduced percent cell viability in the peritoneal macrophages does not necessarily indicate a greater sensitivity of the peritoneal macrophages to the larval products compared to J774 cells. Also, these findings indicate that peritoneal macrophage cells provided more reproducible results for an accurate analysis compared to the J774 cell line.

The results of intracellular amastigote assay suggest that larval saliva and hemolymph suppressed the in vitro growth of amastigote forms of *L. major* at all time points; however, the mechanism of action of the larval products is unclear.

In the present study, larval hemolymph demonstrated higher in vitro anti-amastigote activity and better selectivity than larval saliva. Moreover, the anti-leishmanial effect of treatment with 450 ug/ml larval hemolymph was similar to that of 100 ug/ml glucantime. The findings of the present study also demonstrate that the larval products and glucantime had greater inhibitory effects on intracellular amastigotes than promastigote forms. The PL and SVI values diminished with an increase in concentration and time points.

The results of in vivo experiments in BALB/c mice infected with *L. major* showed that the groups (G1, G4 and G6) of mice treated with either larval saliva or hemolymph had a significantly smaller lesion size compared to the other groups (G2, G3 and G5) and the untreated group (G7).

Interestingly, we observed that the lesion size did not progress in the G4 group mice when the treatment was stopped at week 13, and there was no significant difference between the treated groups (G2, G3 and G5) and the untreated group (G7).

The findings of the present study also show that there was no statistically significant difference in the mean lesion size between male and female mice. Thus, response to treatment with larval saliva and hemolymph was not dependent on sex.

In the in vivo experiments, daily IP administration of larval saliva and hemolymph (G1 and G4) and weekly IL administration of larval hemolymph (G6) were associated with lower parasite burden compared to the untreated control and other treatment groups. Moreover, treatment with daily IP administration of larval hemolymph and saliva (G1 and G4) resulted in a lower parasite burden compared to glucantime (G8).

In this study, we administered larval hemolymph via the intralesional (IL) route so that the chemical compound was completely deposited in the infected footpads, which increased the contact between the larval product and *Leishmania* parasites [44]. Notably, the most effective treatment was the daily IP administration of larval hemolymph (G4). The higher efficacy of daily IP administration of larval hemolymph on lesion size and parasite burden compared with weekly IL administration is probably due to the influence of systemic administration, which could interfere with possible metastasis [45].

## Conclusion

Larval saliva and hemolymph of flies of the species *L. sericata* showed in vitro anti-leishmaniasis activity against promastigote forms of *L. major*. The larval products used in this study did not have any significant cytotoxic effects on either type of macrophage cell line (mouse cell line J774A.1 and peritoneal macrophage cells) for the tested concentrations. Also, the larval products were able to significantly decrease the infection rate of the parasites and the number of intracellular amastigote forms in infected macrophages. Moreover, the larval products of *L. sericata* had significant effects on lesion size and parasitic burden in leishmaniasis models induced by* L.major.* The results of this study suggest that larval saliva and hemolymph of *L. sericata* are potential candidates for leishmanicidal drugs. However, additional studies are recommended to evaluate the effects of these larval products on human subjects. Also, specific components of the larval products and their inhibitory mechanisms on *Leishmania* species are unknown.

## Data Availability

The dataset analyzed during the current study is available from the corresponding author upon reasonable request.

## Abbreviations

CL: Cutaneous leishmaniasis
ACL: Anthroponotic cutaneous leishmaniasis
AmB: Amphotericin B
ES: Excretion and secretion
AMPs: Anti-microbial peptides
SGLs: Salivary gland lysates
SPH-TUMS: School of Public Health, Tehran University of Medical Sciences
IPA: Isopropyl alcohol
PBS: Phosphate-buffered saline
BCA: Bicinchoninic acid
BSA: Bovine serum albumin
CRTSDL: Center for Research and Training in Skin Diseases and Leprosy
NNN: Novy-Macneal-Nicolle
FBS: Fetal bovine serum
RPMI: Roswell Park Memorial Institute
DMEM: Dulbecco’s Modified Eagle Medium
IP: Intraperitoneally
MTT: 3-(4.5-Dimethylthiazol-2-yl)-2, 5-diphenyltetrazolium bromide
DMSO: Dimethyl sulfoxide
OD: Optical density
CC50: Cell cytotoxicity for 50% of cells
IC50: Inhibitory concentration for 50% of parasites
SI: Selectivity index
%I: Infection percentage
%DI: Decreased infection percentage
%V: Viability of amastigote percentage
%DV: Decreased viability of amastigote percentage
GEE: Generalized estimating equation
SVI: Survival index
PL: Parasite load
